# Phosphatidylinositol-5-phosphate 4-kinase gamma accumulates at the spindle pole and prevents microtubule depolymerization

**DOI:** 10.1186/s13008-019-0053-9

**Published:** 2019-08-21

**Authors:** Tz-Chi Lin, Hsiao-Hui Kuo, Yi-Chen Wu, Tiffany S. Pan, Ling-Huei Yih

**Affiliations:** Institute of Cellular and Organismic Biology, Academia Sinica, Taipei, 115 Taiwan

**Keywords:** Mitotic centromere-associated kinesin (MCAK), Microtubule depolymerization, Phosphatidylinositol 4,5-bisphosphate (PIP2), Phosphatidylinositol 5-phosphate 4-kinase type II gamma (PIP4KIIγ), Polo-like kinase (PLK1), Spindle pole

## Abstract

**Background:**

A previous screen of a human kinase and phosphatase shRNA library to select genes that mediate arsenite induction of spindle abnormalities resulted in the identification of phosphatidylinositol-5-phosphate 4-kinase type-2 gamma (PIP4KIIγ), a phosphatidylinositol 4,5-bisphosphate (PIP2)-synthesizing enzyme. In this study, we explored how PIP4KIIγ regulates the assembly of mitotic spindles.

**Results:**

PIP4KIIγ accumulates at the spindle pole before anaphase, and is required for the assembly of functional bipolar spindles. Depletion of PIP4KIIγ enhanced the spindle pole accumulation of mitotic centromere-associated kinesin (MCAK), a microtubule (MT)-depolymerizing kinesin, and resulted in a less stable spindle pole-associated MT. Depletion of MCAK can ameliorate PIP4KIIγ depletion-induced spindle abnormalities. In addition, PIP2 binds to polo-like kinase (PLK1) and reduces PLK1-mediated phosphorylation of MCAK. These results indicate that PIP4KIIγ and PIP2 may negatively regulate the MT depolymerization activity of MCAK by reducing PLK1-mediated phosphorylation of MCAK. Consequently, depletion of PLK1 has been shown to counteract the PIP4KIIγ depletion-induced instability of spindle pole-associated MT and cell resistance to arsenite.

**Conclusions:**

Our current results imply that PIP4KIIγ may restrain MT depolymerization at the spindle pole through attenuating PLK1-mediated activation of MCAK before anaphase onset.

## Background

The spindle microtubule (MT) exhibits highly regulated dynamic instability, with frequent polymerization and depolymerization occurring at both the plus and minus ends. Such dynamism is essential for assembling and positioning the bipolar spindle, searching for and docking with kinetochores, congressing and segregating chromosomes, and governing the spindle checkpoint [1]. The instability of MT dynamics depends heavily on mitotic kinesins, a class of molecular motors, which use the energy from ATP hydrolysis to translocate along the MT or control MT dynamics by facilitating MT polymerization and depolymerization at both ends. Members of the kinesin superfamily (KIF) 2, KIF2A, KIF2B, and MCAK (KIF2C) are MT depolymerizing motors and KIF10 (also known as CENP-E) is a plus-end directed kinesin promoting the elongation of the stabilized MTs [2]. These mitotic kinesins are spatially and temporally regulated to stringently control their localized activity towards both ends of MTs at each stage of mitosis, thereby ensuring mitotic fidelity [3]. In addition, coordination of MT polymerization/depolymerization at both ends to maintain the size and shape of mitotic spindles is also critical for the proper formation and function of the mitotic spindle.

The cellular phosphoinositide signaling system, a complicated network of enzymes and phospholipid messengers, is a crucial regulator for many cellular processes [4]. Phosphatidylinositol 4,5-bisphosphate (PI4,5P2, hereafter referred to as PIP2) either binds to intracellular proteins and directly modulates their subcellular localization and activity or acts as a precursor for the generation of other second messengers. In this way, PIP2 regulates numerous diverse cellular activities, including modulation of the actin cytoskeleton, vesicle trafficking, focal adhesion formation, and several important nuclear events, such as cell cycle progression, apoptosis, chromatin remodeling, transcriptional regulation, and mRNA processing [5–7]. PIP2 is synthesized from phosphatidylinositol 4-phosphate (PI4P) by phosphatidylinositol 4-phosphate 5-kinase type I (PIP5KI), and also from phosphatidylinositol 5-phosphate (PI5P) by phosphatidylinositol 5-phosphate 4-kinase type II (PIP4KII); PIP2 is localized primarily to the plasma membrane, but is also found on secretory vesicles, lysosomes, the endoplasmic reticulum, Golgi, the cytokinesis cleavage furrow, and in the nucleus [8]. Because the members of the PIP5KI and PIP4KII families exhibit specific and distinct subcellular localization patterns, it has been postulated that local increases in PIP2 concentration by a specific kinase member may underlie its diverse signaling functions [5, 8].

It has been demonstrated that members of the PIP5KI and PIP4KII families are specifically phosphorylated at the mitotic stage [9], suggesting that they may contribute to the control of mitotic progression. PIP2 has been demonstrated to bind to tubulin and inhibit MT assembly [10]. PIP2 generated by Skittles, a PIP2 synthesis enzyme in *Drosophila*, is necessary to sustain MT organization for asymmetric transport [11]. Depletion of PIP2 in the plasma membrane perturbs MT organization [12]. Modulation of the activity or localization of PPK-1, a PIP2 synthesis enzyme in *Caenorhabditis elegans*, controls spindle movement by regulating the pulling forces on astral MTs at the one-cell embryo stage [13]. Phospholipase C-γ1, a PIP2 hydrolyzing enzyme, has been reported to interact with β-tubulin, and to thereby increase its prevalence on spindle poles during mitosis [14]. In addition, phosphatidylinositol 4-phosphate 5-kinase alpha (PIP5KIα) has recently been demonstrated to regulate neuronal MT depolymerase kinesin KIF2A and suppress the elongation of axon branches [15]. These studies reveal that MT dynamics may be regulated by PIP2 generated at distinct cellular localizations by a specific member of the two phosphatidylinositol phosphate kinase (PIPK) families at different cell cycle stages, and that PIP2/PIPK may control MT dynamics through modulating MT-depolymerizing kinesin activities.

Previously, screening of a human kinase and phosphatase shRNA library to select genes that mediate arsenite induction of spindle abnormalities resulted in the identification of PIP4KIIγ [16], indicating that PIP4KIIγ may be involved in the control of spindle assembly through regulating MT dynamics. In the current study, the role of PIP4KIIγ in the assembly of the mitotic spindle was investigated. Our results show that PIP4KIIγ may restrain MT depolymerization at the spindle pole through attenuating PLK1-mediated phosphorylation of MCAK before the onset of anaphase.

## Results

### PIP4KIIγ accumulates at the spindle pole and associates with α-tubulin

**Fig. 1:**
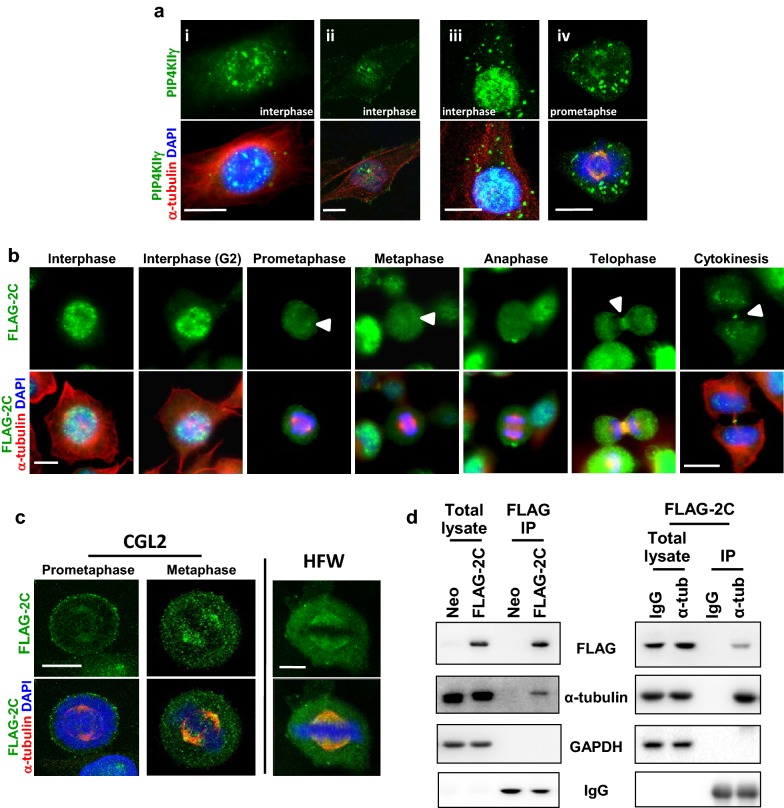
PIP4KIIγ accumulates at the spindle pole and the distal ends of spindle fibers. **a**–**c** Representative images of the cellular distribution of PIP4KIIγ. **a** CGL2 cells were fixed and stained for PIP4KIIγ (green) with commercial antibodies form Sigma (**a-**i and ii) and from GeneTex (**a-**iii and iv) and α-tubulin (red). **b**, **c** Representative images of CGL2 cells stably or human fibroblasts (HFW) transiently expressing FLAG-tagged PIP4KIIγ (FLAG-2C) were fixed and stained for FLAG-2C (green) and α-tubulin (red). Nuclei or chromosomes were counterstained with DAPI (blue). Images were obtained using a fluorescence microscope (**a**, **b**) or an upright confocal microscope (**c**). Scale bar, 10 μm. **d** FLAG-tagged PIP4KIIγ associates with α-tubulin. Left, cells stably expressing empty vector (Neo) or FLAG-2C were subjected to immunoprecipitation (IP) using anti-FLAG antibodies. Right, cells stably expressing FLAG-2C were subjected to IP using mouse IgG or anti-α-tubulin (α-tub) antibodies. The immunoprecipitates were then analyzed by immunoblotting with the indicated antibodies

Cellular localization of PIP4KIIγ was examined in CGL2 cells by immunofluorescence staining with two commercial antibodies. The results showed that PIP4KIIγ was stained in a puncta-like structure scattering in the cytoplasm and nucleus during interphase (Fig. 1a-i–iii). PIP4KIIγ became accumulated at the spindle pole and the distal end of spindle MT along with many puncta-like structures in the cytoplasm of a prometaphase cell (Fig. 1-iv). Cellular localization of PIP4KIIγ was also examined in CGL2 cells stably expressing the FLAG-tagged PIP4KIIγ (FLAG-2C). Figure 1b shows that FLAG-tagged PIP4KIIγ was mainly present in the nuclei during interphase, accumulated at the spindle pole and the distal end of spindle MT during prometaphase and metaphase, became diffused at the onset of anaphase, and moved to the cleavage furrow during cytokinesis. The emergence of PIP4KIIγ at the spindle pole from prometaphase to metaphase was also verified by confocal microscopy (Fig. 1c). Similar results were obtained in a human diploid fibroblast cell line transiently expressing the FLAG-tagged PIP4KIIγ (Fig. 1b, right panel), indicating that the spindle pole accumulation of PIP4KIIγ might be p53-independent. In addition, co-immunoprecipitation studies of the control (Neo) and cells stably expressing FLAG-tagged PIP4KIIγ (FLAG-2C) revealed that a small fraction of α-tubulin was pulled down with the overexpressed FLAG-tagged PIP4KIIγ (Fig. 1d left). A small fraction of FLAG-tagged PIP4KIIγ was also pulled down with α-tubulin (Fig. 1d right). These results suggest that PIP4KIIγ may interact with MTs at and around the spindle pole.

### PIP4KIIγ is required for the assembly of bipolar spindles

**Fig. 2:**
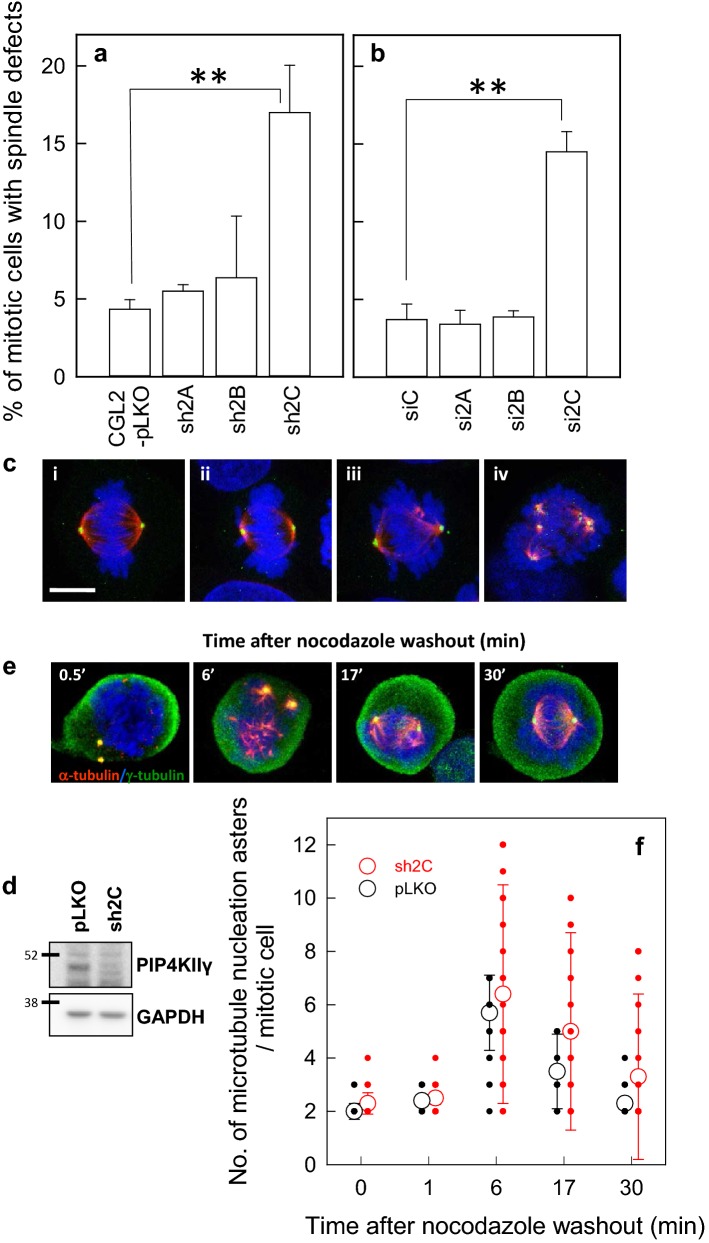
PIP4KIIγ is required for assembly of bipolar spindles. **a**, **b** Depletion of PIP4KIIγ induces defects in spindle assembly. CGL2 cells depleted of PIP4KIIα, β, or γ using specific shRNA (**a**) or siRNA (**b**) were then fixed and stained for mitotic spindles with antibodies against α-tubulin and γ-tubulin. The percentage (mean ± SD) of mitotic cells containing abnormal mitotic spindles was determined using at least 500 mitotic cells from three independent experiments. **p < 0.01 by Student’s *t* test compared to the control depleted cells. **c** Representative images of the mitotic spindle immunofluorescence-stained for α-tubulin (red), γ-tubulin (green), and chromosome (blue) in PIP4KIIγ-depleted culture. i, the normal bipolar spindle. ii and iii, the bipolar spindle with mis-aligned chromosomes. iv, the multipolar spindle. **d** The depletion efficiency of PIP4KIIγ by specific shRNA. **e** Representative images of the mitotic spindle after nocodazole treatment and washout. The control (pLKO) or cells depleted of PIP4KIIγ (sh2C) were treated with 3 μM Nocodazole for 3 h. Nocodazole was then completely washed out, and the cells were incubated in drug-free medium for the indicated time before being immediately fixed and immunostained for α-tubulin (red) and γ-tubulin (green). The chromosomes were counterstained with DAPI (blue). **f** The number of MT asters in cells. The results are the distribution and mean ± SD of the aster numbers in at least 250 mitotic cells for each condition, as determined from three experiments

We proceeded to examine whether PIP4KIIγ regulates the assembly of mitotic spindles. The integrity of mitotic spindles was examined in CGL2 cells depleted of PIP4KIIα, β, or, γ via lentivirus-based shRNA transduction (Fig. 2a) or the use of isoform-specific siRNA (Fig. 2b). The depletion efficiency of each construct has been previously demonstrated [16]. Figure 2a, b showed that the depletion of PIP4KIIγ, but not PIP4KIIα or PIP4KIIβ, resulted in a considerable increase of mitotic cells containing mitotic spindles with an abnormally organized bipolar (Fig. 2c-ii, iii) or a multipolar (Fig. 2c-iv) pattern. These aberrant mitotic cells contained well-condensed and mis-aligned chromosomes (Fig. 2c-ii–iv), and were rarely at anaphase. These results confirm that PIP4KIIγ is required for the assembly of a bipolar mitotic spindle for proper chromosome segregation. Thus PIP4KIIγ may have a role distinctive to that of other PIP4KIIs, and played a role in spindle assembly.

**Fig. 3:**
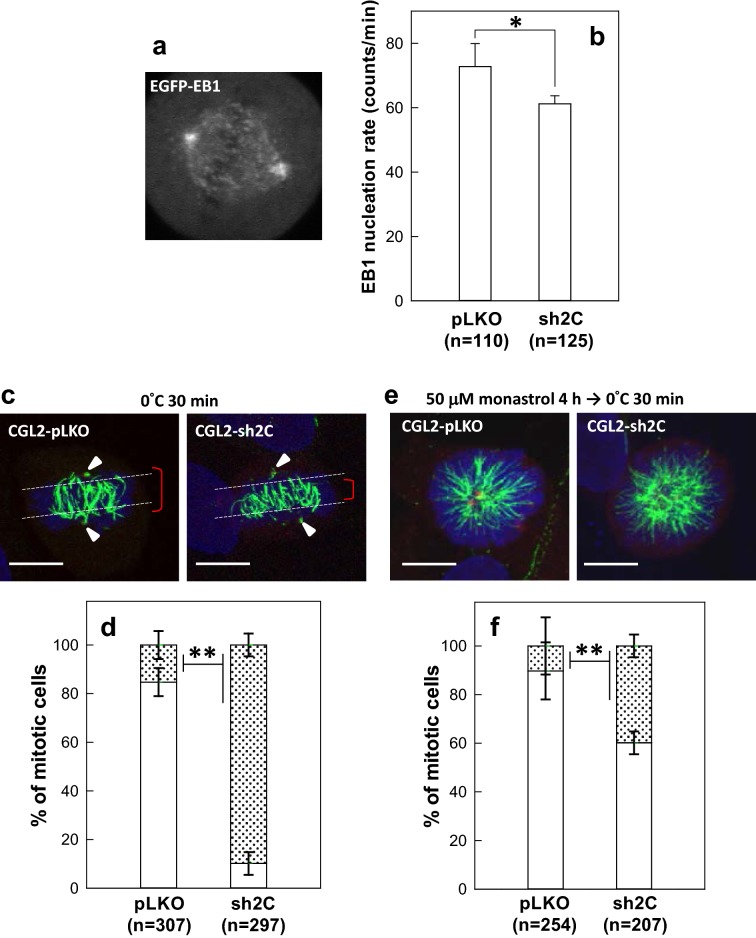
Spindle pole-associated MTs are less stable in PIP4KIIγ-depleted mitotic cells. **a** A representative image frame of a time-lapse sequence from a cell stably expressing EGFP-EB1 (HeLa-EB1-GFP). **b** Depletion of PIP4KIIγ reduces the nucleation of EGFP-EB1 comets. The control depleted (pLKO) or PIP4KIIγ-depleted (sh2C) HeLa-EGFP-EB1 cells were subjected to time-lapse imaging under a confocal microscope as described in “Methods” section. Typically, each comet was visible for three to five frames. Results are the mean ± SD of EB1 comets nucleated from at least 100 spindle poles in three independent experiments. *p < 0.05 by Student’s *t* test compared to the control (pLKO). **c**, **d** Depletion of PIP4KIIγ shortens cold-resistant K-MT. Representative images of the mitotic spindle after cold treatment (**c**). The control (pLKO) or PIP4KIIγ-depleted (sh2C) CGL2 cells were subjected to cold treatment for 30 min before being stained for mitotic spindles as described in Fig. 2c. Arrowheads indicate the spindle poles. The percentages of mitotic cells with long (blanked bar) or short (dotted bar) cold-resistant K-MTs (**d**). **e**, **f** Depletion of PIP4KIIγ disorganizes the spindle pole-associated cold-resistant K-MT. Representative images of the mitotic spindle after monastrol and cold treatment (**e**). The control (pLKO) or PIP4KIIγ-depleted (sh2C) CGL2 cells were first treated with 50 μM monastrol for 4 h to prevent centrosome separation and then subjected to cold treatment for 30 min before being stained for mitotic spindles as described in Fig. 2b. The percentages of mitotic cells with well-organized (blanked bar) or disorganized (dotted bar) spindle pole-associated cold-resistant K-MT (**f**). Results are the mean ± SD of at least 250 mitotic cells from three independent experiments. **p < 0.01 by Student’s *t* test compared that in the control. Scale bar, 10 μm

**Fig. 4:**
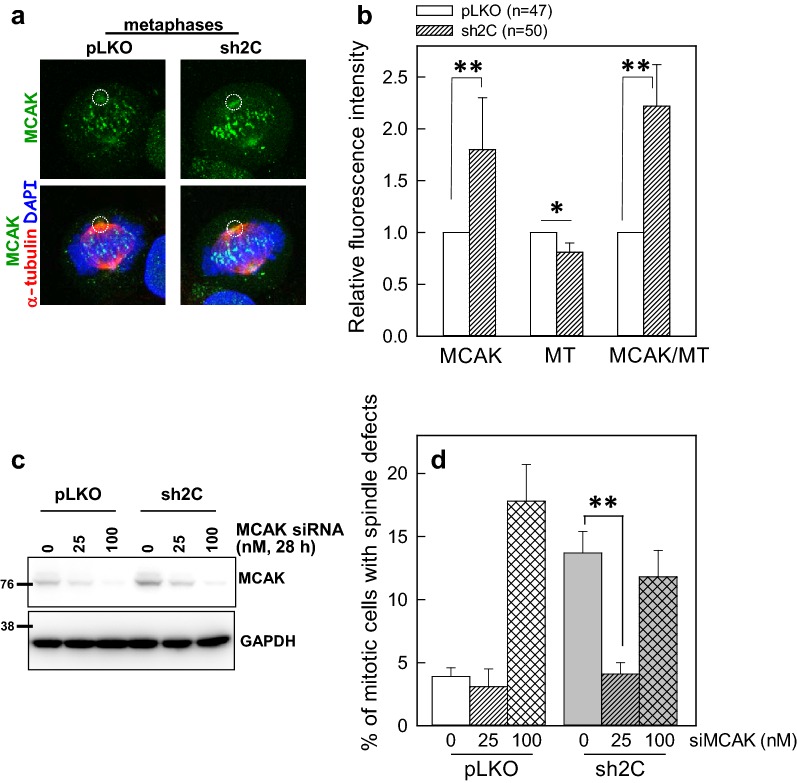
Depletion of PIP4KIIγ enhances MCAK accumulation at the spindle pole. **a** Representative images of cells immunostained for MCAK (green) and α-tubulin (red). The control or PIP4KIIγ-depleted CGL2 cells were fixed and stained for MCAK and α-tubulin. **b** Relative intensities of MCAK or α-tubulin at the spindle pole. Results are the mean ± SD of at least 200 mitotic cells from three independent experiments. *p < 0.05 and **p < 0.01 by Student’s *t* test as compared that in the control. **c** Depletion of MCAK by siRNA transfection. The control- or PIP4KIIγ-depleted CGL2 cells were transfected with 0, 25, or 100 nM MCAK-specific siRNA as described in “Methods” section. Twenty-eight hours after transfection, the cells were subjected to immunoblotting to examine the depletion efficiency of MCAK. **d** Depletion of MCAK prevents the formation of spindle defects in PIP4KIIγ-depleted cells. The percentage (mean ± SD) of mitotic cells containing abnormal mitotic spindles was determined using at least 500 mitotic cells from three independent experiments. **p < 0.01 by Student’s *t* test compared to cells that were not transfected with siRNA

**Fig. 5:**
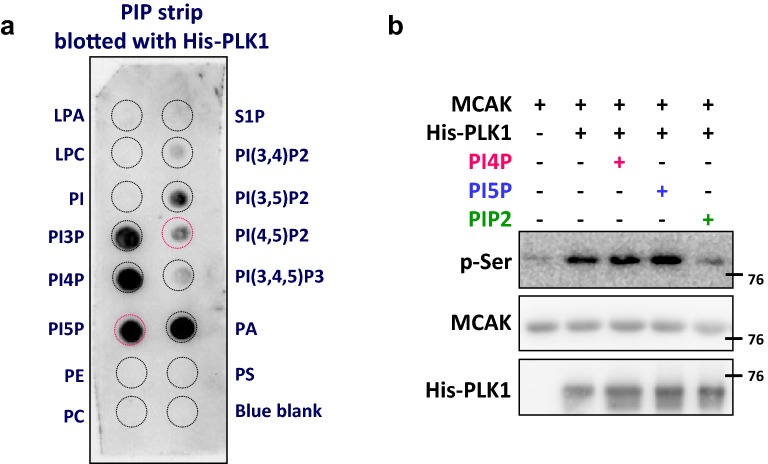
PIP2 binds to PLK1 and reduces PLK1-mediated phosphorylation of MCAK. **a** A representative blot showing the binding of PLK1 to PIPs. The PIP strip was incubated with recombinant human PLK1. The binding of PLK1 to each PIP was then detected and visualized as described in “Methods” section. **b** PIP2 reduces the phosphorylation of MCAK by PLK1. The MCAK immunoprecipitated from cells was incubated with recombinant PLK1, and MCAK phosphorylation was verified by immunoblotting with anti-phospho-serine antibody as described in “Methods” section

**Fig. 6:**
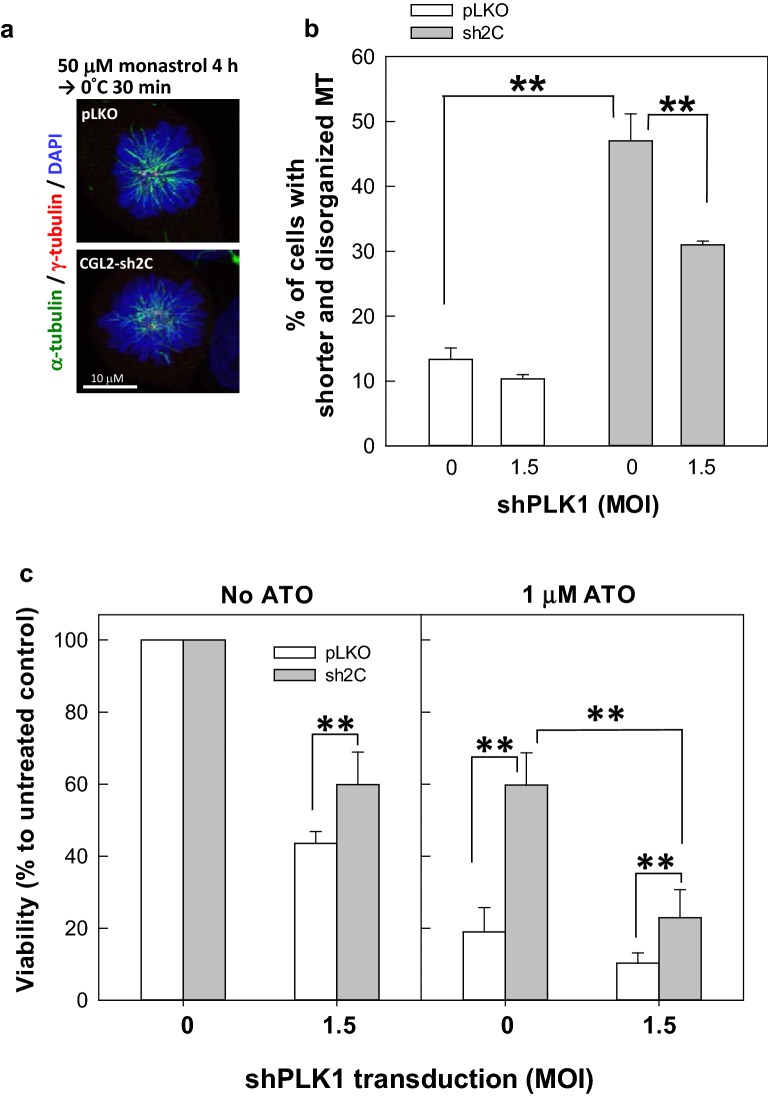
PLK1 depletion antagonizes PIP4KIIγ depletion-induced MT instability. **a**, **b** Down-regulation of PLK1 ameliorates PIP4KIIγ depletion-induced instability of spindle pole-associated MT. Representative images of the mitotic spindle after monastrol and cold treatment (**a**). The control (pLKO) or PIP4KIIγ-depleted (sh2C) CGL2 cells were first transduced with virion containing PLK1-specific shRNA at the indicated MOI. Forty-eight hours after transduction, the cells were seeded onto coverslips for another 20 h before monastrol and cold treatment as described in Fig. 3e. The percentages of mitotic cells with disorganized and shorter spindle pole-associated cold-resistant K-MT (**b**). Results are the mean ± SD of at least 250 mitotic cells from three independent experiments. **p < 0.01 by Student’s *t* test. **c** Down-regulation of PLK1 represses the PIP4KIIγ depletion-induced cell resistance to arsenic trioxide (ATO). The control (pLKO) or PIP4KIIγ-depleted (sh2C) CGL2 cells were first transduced with virion containing PLK1-specific shRNA at the indicated MOI. Forty-eight hours after transduction, the cells were seeded for another 20 h before being treated with 0 or 1 μM ATO for 72 h. Cell viability was then determined. Results are the mean ± SD from three independent experiments. **p < 0.01 by Student’s *t* test

We subsequently examined how PIP4KIIγ regulates the assembly of mitotic spindles. MT regrowth after nocodazole washout was followed in the control (pLKO) and cells depleted of PIP4KIIγ (sh2C). Figure 2d showed the depletion efficiency of PIP4KIIγ. Under 3 μM nocodazole treatment, the MTs of mitotic spindles in both cells were completely disassembled, leaving two γ-tubulin spots in the mitotic cell (Fig. 2e-0.5′). In the control pLKO cells, MT asters (as shown by the immunostaining of α-tubulin, red) formed immediately after nocodazole release, and their number increased during the first 6 min (Fig. 2e-6′), then progressively fused together with the centrosomal aster (as indicated by the co-immunostaining of α-tubulin and γ-tubulin) to organize a bipolar spindle at 17 and 30 min (Fig. 2e-17′ and 30′). Control pLKO cells contained 2 MT asters at 0.5–1 min after nocodazole release, and had an average of six asters per cell at 6 min. At 17 and 30 min after nocodazole release, the number of asters per cell was only three and two respectively, reflecting the reorganization of the bipolar spindle (Fig. 2f). In PIP4KIIγ-depleted cells (sh2C), MT asters also appeared at a similar rate to that in the control cells, but the number of aster at each time point after nocodazole release was more diverse, as revealed by the magnitude of deviation bars (Fig. 2f). These results indicate that depletion of PIP4KIIγ does not interfere with the formation of the centrosomal and non-centrosomal MT asters, but may alter the stability of aster MTs, resulting in disruption of the coalescence of MT asters and delayed re-organization of bipolar spindles.

### The spindle pole-associated MT is less stable in PIP4KIIγ-depleted mitotic cells

To determine how PIP4KIIγ depletion alters the reassembly of the mitotic spindle, we performed live-imaging of EGFP-tagged EB1, a microtubule tip-binding protein, and subsequently calculated the rate of EB1-positive comet emanation from the mitotic centrosome (Fig. 3a). We observed that the rate of EGFP-EB1 comet nucleation from the spindle pole in PIP4KIIγ-depleted cells was significantly reduced compared to that in the control (Fig. 3b), indicating that PIP4KIIγ depletion may hamper MT nucleation and/or polymerization from the spindle pole.

The effect of PIP4KIIγ depletion on the stability of the spindle MT was then examined by exposing mitotic cells to cold and/or treating them with monastrol. Under cold treatment, the kinetochore MTs (K-MTs) remain relatively stable, whereas most other MTs undergo depolymerization [17]. During metaphase in the control CGL2-pLKO cells, cold treatment for 30 min resulted in disassembly of most MT, leaving the K-MTs bundled together in mitotic cells as expected (Fig. 3c left). The cold-resistant K-MTs were much shorter in 90% of CGL2-sh2C in metaphase (Fig. 3c right and d dotted bar), and the distal part of K-MTs (Fig. 3c, near the arrowhead indicated spindle pole) mostly disappeared (Fig. 3c right). The presence of cold-stable K-MT suggested that the kinetochore attachment site might not be altered in PIP4KIIγ-depleted mitotic cells. These results indicate that the distal part of K-MT in PIP4KIIγ-depleted mitotic cells was more sensitive to cold treatment than that in the control. To confirm this, the K-MTs were examined in cells treated with monastrol, an Eg5 inhibitor that prevents centrosome separation, before cold treatment. As expected, the K-MTs were much shorter, and their minus ends were disorganized around the spindle pole (Fig. 3e right) in 40% mitotic CGL2-sh2C cells (Fig. 3f dotted bar) as compared to the control mitotic cells. These results indicate that the minus ends of spindle pole-associated MTs were less stable and depolymerized much faster in PIP4KIIγ-depleted cells.

### Depletion of PIP4KIIγ enhances MCAK accumulation at the spindle pole

Since PIP4KIIγ is localized at the spindle pole and its depletion results in less stable spindle pole-associated MT, we hypothesized that depletion of PIP4KIIγ may induce depolymerization of MT at the spindle poles. As members of the KIF2 are MT depolymerizing motors, we examined their distribution by immunofluorescence staining in control and PIP4KIIγ-depleted cells. The results showed that the accumulation of MCAK (Fig. 4a), but not KIF2A or KIF2B (data not shown), at the spindle pole was considerably elevated in the PIP4KIIγ-depleted mitotic cells (Fig. 4b). MCAK staining intensity at the kinetochores also increased in mitotic sh2C cells. In addition, the intensity of α-tubulin around the spindle pole were slightly but significantly decreased during metaphase in the PIP4KIIγ-depleted cells (Fig. 4b). These results indicate that depletion of PIP4KIIγ may elevate MCAK accumulation and enhance MT depolymerization at the spindle pole.

The role of enhanced accumulation of MCAK at the spindle pole in PIP4KIIγ-depleted cells was then verified by siRNA-mediated depletion of MCAK. Figure 4c shows that cellular MCAK levels were efficiently reduced to 25% and less than 5% by treating cell with 25 and 100 nM MCAK-specific siRNA respectively as compared with that in cells untreated with the siRNA, in both the control (pLKO) and PIP4KIIγ-depleted (sh2C) cells. The effect on MCAK depletion on spindle assembly was then examined in these cells: moderate depletion of MCAK by 25 nM siRNA in pLKO cells did not induce significant spindle abnormalities (Fig. 4d). Extreme depletion of MCAK by 100 nM siRNA resulted in a considerable increase of mitotic cells with abnormal spindles, consistent with a previous report showing that MCAK is required for the assembly of mitotic spindles [18]. Consistent with the result shown in Fig. 2a, PIP4KIIγ depletion induced the formation of abnormal mitotic spindles (Fig. 4d). Strikingly, a moderate decrease of MCAK in PIP4KIIγ-depleted cells could significantly reduce the formation of abnormal spindles (Fig. 4d). These results indicate that PIP4KIIγ depletion-induced spindle defects can be rescued by moderate depletion of MCAK, and suggest that the elevated MCAK activity may be involved in induction of spindle abnormalities in the PIP4KIIγ-depleted cells.

### PIP2 binds to PLK1 and reduces PLK1-mediated phosphorylation of MCAK

MCAK was previously demonstrated to be phosphorylated by PLK1 to induce depolymerization of spindle MT [19]. Since PLK1 has been described in a screen for PIP2-interacting proteins [20], we examined whether PIP interacts with PLK1 and regulates the MT depolymerization activity of MCAK. Figure 5a shows that PLK1 binds strongly to PI3P, PI4P, and PI5P, and moderately to PI(3,5)P2 and PIP2.

Our in vitro phosphorylation assay revealed that addition of PLK1 substantially increased MCAK serine phosphorylation (Fig. 5b). The level of PLK1-induced serine phosphorylation of MCAK was not significantly changed in the presence of PI4P or PI5P, but dramatically decreased in the presence of PIP2. These results indicated that the binding affinity of PIP2 to PLK1 is low, but that PIP2 can effectively reduce the phosphorylation activity of PLK1 towards MCAK. These in vitro studies indicate that interaction of PIP2 with PLK1 may reduce PLK1-mediated phosphorylation of MCAK.

### PLK1 depletion antagonizes PIP4KIIγ depletion-induced MT instability

The role of PLK1 in PIP4KIIγ depletion-induced MT depolymerization was subsequently verified by examining the spindle pole-associated MTs, following depletion of PLK1 in cells stably depleted of PIP4KIIγ. In non-PIP4KIIγ-depleted cells, a moderate depletion of PLK1 did not significantly change the length or organization of the spindle pole-associated MTs (Fig. 6a upper panel and b). Consistent with the result shown in Fig. 3f, PIP4KIIγ depletion resulted in a considerable increase in the number of cells in which the spindle pole-associated MTs exhibited shorter and disorganized MT ends (Fig. 6a lower panel and b). This increase was significantly reduced by depletion of PLK1 (Fig. 6b). Thus, PLK1 depletion counteracts the MT instability observed in PIP4KIIγ-depleted cells. These results indicate that PIP4KIIγ may confine the PLK1 activity at the spindle pole. In addition, PIP4KIIγ-depleted cells were more resistant than the non-PIP4KIIγ-depleted cells to PLK1 depletion-induced cell death (Fig. 6c left panel). Previously, we demonstrated that cells depleted of PIP4KIIγ are more resistant to arsenic trioxide [16]. The current results showed that this resistance was also repressed by PLK1 depletion (Fig. 6c right panel). These results confirm that the cellular effects of PIP4KIIγ depletion can be alleviated by PLK1 depletion.

## Discussion

One of the important regulatory processes involved in the initiation of chromosome segregation is the depolymerization of spindle pole-associated MT minus ends, which induces a change in MT dynamics that enables the movement of sister chromatids to spindle poles at the onset of anaphase. However, the cellular machinery that controls the performance and organization of MT minus ends remains one of the least well-understood features of MT dynamics. In this study, we provide multiple lines of evidence that PIP4KIIγ may be a component of the machinery that regulates MT dynamics and restrains MT depolymerization at the spindle pole before the onset of anaphase.

As a negative regulator of MT depolymerization at the spindle pole at early mitosis, PIP4KIIγ association with the spindle pole may be delicate and stringently regulated. Although PIP4KIIγ has not been experimentally verified to localize at the centrosome (as was reported in a database for the temporal and spatial localization of proteins in distinct subcellular positions [21]), our immunofluorescence staining and immunoprecipitation findings indicate that PIP4KIIγ accumulates at the spindle pole during mitosis. In addition, it was reported the enzymes that are involved in PIP2 processing, which include phospholipase C-γ1, phosphoinositide-3-kinase catalytic α polypeptide, and protein kinase C-ε and -θ, accumulate at the centrosome [21]. However, we failed to detect PIP2 accumulation at the spindle pole by immunofluorescence staining with a commercial anti-PIP2 antibody (MA3-500, Invitrogen, data not shown). This may be because the spindle pole-associated PIP2 was disrupted by the fixative used in sample preparation for immunofluorescence staining, or because PIP2 is embedded deeply in the pericentriolar materials, and is thus not recognized by the antibody. Alternatively, these results may also imply that the spindle pole-associated PIP2 is highly dynamic and rapidly processed.

We also demonstrated that the association between PIP4KIIγ and the spindle pole is temporally regulated during mitosis, with PIP4KIIγ accumulating at the spindle pole upon entering mitosis and disappearing at the onset of anaphase. This regulation might be controlled by cyclin-dependent kinase 1 (CDK1), as inhibition of CDK1 at metaphase results in an immediate loss of PIP4KIIγ from the spindle pole (data not shown). As in the case of other spindle pole proteins, PIP4KIIγ may depend on CDK1, the master mitotic kinase, for its spindle pole localization and function. After initiation of anaphase, CDK1 activity gradually decreases, and thus may be unable to maintain the spindle pole accumulation of PIP4KIIγ. Our finding that depletion of PIP4KIIα or PIP4KIIβ did not induce defects in mitotic spindle assembly indicates that these PIPKs may not be involved in the regulation of spindle assembly. It has been demonstrated that PIP5KIγ targets to the interphase centrosome and negatively regulates centriole duplication by binding and inhibiting PLK4 activity [22]. PIPKs are known to regulate diverse cellular functions through strict regulation of their spatial and/or temporal expression at specific cellular compartments. Our results thus indicate a specific function of PIP2 and PIP4KIIγ at the spindle pole before the onset of anaphase. Further investigations are warranted to elucidate the mechanism underlying PIP4KIIγ accumulation at the spindle pole.

Our results showed that the negative regulation of MT depolymerization via PIP2 and PIP4KIIγ seems to act through preventing the complete activation of PLK1. PLK1 was described in a screen for PIP2-interacting proteins [20]. PLK1 is characterized by its N-terminal kinase domain (KD) and a C-terminal polo-box domain (PBD); the KD and PBD exert mutual inhibitory effects [23]. The reported putative PIP2 binding motif (aa 134–146) of PLK1 [20] is within the N-terminal KD, and contains one (Ser137) of the two main activating phosphorylation sites of the KD [23, 24]. It has been shown that the KD stabilizes a conformation of the PBD that is less capable of interacting with phosphorylated targets, and that phosphorylation at Ser137 interferes with the inhibitory interaction of the PBD on the KD [25]. Our results revealed that PLK1 interacts strongly with PI4P and PI5P, and moderately with PIP2. However, only PIP2 can significantly reduce the phosphorylation of MCAK by PLK1. Thus, the binding of PIP2 might specifically attenuate the activating phosphorylation of PLK1, and thereby prevent its complete activation and interaction with target proteins. Accumulation of PIP4KIIγ at the spindle pole before anaphase may therefore induce the generation of PIP2 at the spindle pole, where it restrains MT depolymerization by preventing complete activation of PLK1 at specific mitotic stages.

PLK1 has been demonstrated to phosphorylate MCAK and promotes its MT depolymerase activity during the early phases of mitosis [19, 26]. MCAK is localized to inner centromeres, kinetochores, and spindle poles of mitotic cells, and its subcellular localization and activity are controlled by phosphorylation [27]. The role of MCAK function in the error-correction at the kinetochore and MT plus ends is well-documented [28]. MCAK also accumulates at the spindle poles [29] and it has MT minus-end depolymerase activity controlling the dynamics of spindle pole-associated MT [30, 31]. It has also been demonstrated that restriction of the MCAK depolymerase activity at spindle pole is essential for assembly of normal functioning metaphase spindles and anaphase onset [31]. Our results demonstrated that a fraction of MCAK is localized to the spindle pole, and that PIP4KIIγ-depleted mitotic cells show enhanced spindle pole localization of MCAK. Our in vitro studies also demonstrated that PLK1 phosphorylates MCAK and PLK1-mediated phosphorylation of MCAK was considerably reduced in the presence of PIP2. Thus, MCAK may be the downstream effector of PLK1 responsible for the enhanced MT depolymerization observed in PIP4KIIγ-depleted mitotic cells.

It is known that inhibition of PLK1 induces mitotic cell death due to an inability of cells to undergo centrosome separation [32, 33]. In our study, we noticed that depletion of PLK1 also induces cell death mainly via induction of monopolar spindles and mitotic arrest (data not shown). Interestingly, the PLK1 depletion-induced cell death can be ameliorated in PIP4KIIγ-depleted cells. In addition, PIP4KIIγ-depleted cells were also more resistant to arsenic trioxide, a mitosis-disrupting drug that may induce MT stabilization and therefore attenuate the dynamic instability of spindle MT [34, 35], compared to the non-PIP4KIIγ-depleted cells [16]. These results indicate that depletion of PIP4KIIγ may enhance PLK1 activity and MT depolymerization and hence antagonize the cellular effects-induced by PLK1 depletion or arsenic trioxide and confirm that PIP4KIIγ may confine PLK1 function and thereby prevent MT depolymerization.

## Conclusions

In this study, we employed several methods to delineate the role of PIP4KIIγ in maintaining the stability of spindle pole-associated MT before the onset of anaphase. First, loss of PIP4KIIγ was observed to hamper the ability of cells to reassemble a bipolar spindle after nocodazole washout. Second, the spindle MTs, especially those near the spindle pole, were found to be more sensitive to cold treatment, and thus less stable, in mitotic cells lacking PIP4KIIγ. Third, depletion of PIP4KIIγ significantly reduced MT nucleation, as indicated by the reduced rate of EB1 comet emanation from the spindle pole. These results imply that spindle pole-associated MT was less stable in mitotic cells lacking PIP4KIIγ. In addition, we also demonstrated that the enhanced MACK activity at the spindle pole may mediate PIP4KIIγ depletion-induced instability of spindle pole-associated MT. Furthermore, we also provide evidence that PIP2 may interact with PLK1 and reduce PLK1-mediated phosphorylation and activation of MCAK. Since PIP4KIIγ localizes to the spindle pole during prophase to metaphase, these results indicate that PIP4KIIγ-mediated generation of PIP2 may act to restrict MT depolymerization at the spindle pole before anaphase onset. It is known that depolymerization of spindle pole associated-MT minus ends provides a driving force for chromosome segregation at anaphase [36, 37]. Our results thus imply that PIP2 and PIP4KIIγ may be the components of the machinery that regulate the dynamics of spindle pole-associated MT minus ends.

## Methods

### Cell culture

CGL2 cells were cultured as previously described [16]. HeLa cells stably expressing EGFP-EB1 were established by transfecting cells with the expression vector pEGFP-N1-EB1 (a gift from Dr. Tim Mitchison, Addgene plasmid # 12345) and selecting under G418 [38]. CGL2 cells stably depleted of PIP4KIIγ (sh2C) were previously described [16]. CGL2 cells overexpressing the FLAG-tagged PIP4KIIγ were established by transducing cells with virions containing PIP4KIIγ cDNA and then selecting under 1 mg/ml neomycin (Invitrogen, Carlsbad, CA). Full-length PIP4KIIγ cDNA was amplified by PCR using primers in which FLAG tag and restriction sites for *Eco*RI and *Xho*I had been created (5′- GGAATTCGCCACCATGGATTACAAGGATGACGACGATAAGATGGCGTCCTCCTCGGTC-3′ and 5′-CGGGATCCTTAGGCAAAGATGTTGGTAATAAAATC -3′; GenBank accession number NM_001146258). Products were digested with *Eco*RI and *Xho*I and then subcloned into the corresponding sites of the retroviral pFB-Neo vector (Stratagene/Agilent Technologies, La Jolla, CA). Virus particles containing the PIP4KIIγ expression construct were generated as previously described [39]. Cell viability was examined as described [39].

### Depletion of PIP4KII, MCAK, or PLK1

Depletion of each member of the PIP4KII family was achieved by transfecting cells with small interfering RNAs (siRNAs) or by transducing cells with VSV-G-pseudotyped lentivirus-based short hairpin RNA (shRNA), as previously described [16]. The siRNAs specific to each PIPK isoform were obtained from Thermo Scientific Dharmacon (On-Target plus SMART pool; Lafayette, CO). The shRNAs specifically targeting PIP4KIIα (TRCN10988), PIP4KIIβ (TRCN196947), PIP4KIIγ (TRCN37719), or PLK1 (TRCN121325) were obtained from the National RNAi core Facility Platform (Genomic Research Center, Academia Sinica). The shRNA-containing virions were prepared and collected, and depletion of each PIPK or PLK1 were carried out as previously described [16]. For depletion of MCAK, cells were transfected with specific siRNAs (On-Target plus SMART pool, Thermo Scientific Dharmacon) as previously described [16].

### Immunoblotting and immunoprecipitation

Cell lysis, immunoblotting, and immunoprecipitation were carried out as previously described [38, 39]. For immunoprecipitation, cells were lysed with RIPA buffer for 30 min on ice. Alpha-tubulin or FLAG-tagged PIP4KIIγ was immunoprecipitated with specific antibodies. Immunoprecipitated complexes were then subjected to immunoblot analysis. Specific proteins were immunoprecipitated or detected using antibodies against MCAK (Cytoskeleton, Denver, CO), PLK1 (Invitrogen, Grand Island, NY), and FLAG, phospho-serine, α-tubulin, and γ-tubulin (Sigma, St. Louis, MO). Beta-actin or GAPDH was detected with anti-β-actin antibody (Chemicon, Temecula, CA) or anti-GAPDH antibody (Genetex, Hsinchu, Taiwan), respectively, for use as loading controls.

### Immunofluorescence staining and fluorescence microscopy

Cells were seeded onto coverslips, incubated for 20 h prior to drug treatments, and then fixed in PTEMF buffer (20 mM PIPES, 4% para-formaldehyde, 0.2% Triton-X, 10 mM EGTA, and 1 mM MgCl_2_) for 15 min. For depletion of each PIPK, cells were transfected with siRNA targeting each PIP4KII or transduced with virion containing shRNA of each PIP4KII for 48 h, and were then seeded onto coverslips for another 20 h before fixation. Immunostaining, observation and imaging of the stained cells, and image analysis were carried out as previously described [38]. Anti-FLAG (Sigma), anti-α-tubulin (Sigma), anti-γ-tubulin (Sigma), anti-MCAK (Cytoskeleton, Inc. Denver, CO), and anti-PIP4KIIγ (WH0079837M1/Sigma and GTX119713/GeneTex) antibodies were used for immunostaining. Alexa Fluor 488-, 568-, or 633-conjugated goat anti-mouse and rabbit IgG were obtained from Invitrogen. For measurement of the intensity of MCAK or α-tubulin, exposure time was set and kept constant throughout each independent experiment. The region of interest (ROI) was defined by drawing a circle enclosing the mitotic centrosome. The intensities of MCAK or α-tubulin within ROI were determined by measuring average pixel intensity with MetaMorph COMPLETE software (Molecular Device, Sunnyvale, CA). Background fluorescence (based on a circle of corresponding size in an adjacent region) was subtracted from each measurement.

### Analysis of spindle MT reassembly

The effect of PIP4KIIγ on spindle MT polymerization was assessed using nocodazole washout assay. Control CGL2 (pLKO) or CGL2 cells depleted of PIP4KIIγ (sh2C) seeded on coverslips in 6-well plates were subjected to nocodazole treatment (3 μM for 3 h). Nocodazole was then washed out and replaced with warmed media, followed by incubation at 37 °C for the indicated time. The cells were fixed and immunostained with antibodies against α- and γ-tubulin after the indicated period of re-growth as previously described [38]. The number of MT asters was counted under a fluorescent microscope (Zeiss Axioplan 2 Imaging MOT).

### Analysis of MT nucleation

MT nucleation was visualized by live-imaging of EGFP-EB1 [40]. EB1 is a MT tip-binding protein. The EGFP-tagged EB1 is frequently used to determine the rates of MT nucleation form the spindle pole by counting the number of EB1-GFP comets emerging from the centrosome over time. HeLa cells stably expressing EGFP-EB1 were first depleted of PIP4KIIγ and then seeded in chambered coverslips (μ-slide 4 well, ibidi GmbH, Germany). The cells were then monitored and imaged under an inverted confocal microscope (Leica TCS-SP5) as previously described [38]. Time lapse images were then compiled and analyzed using MetaMorph software. EGFP-EB1 comets emanating from the spindle pole and visible in a consecutive series of three to five frames were identified and counted.

### Analysis of spindle MT stability

The effect of PIP4KIIγ on spindle MT stability was assessed by cold treatment. Control CGL2 (pLKO) or CGL2 cells depleted of PIP4KIIγ (sh2C) were seeded onto coverslips in 6-well plates. The plates were immersed in ice-cold water for 30 min, and then the cells were immediately fixed in PTEMF buffer, stained with antibodies against α-tubulin and γ-tubulin, and imaged using a confocal microscope (Leica TCS-SP5). Alternatively, cells were first treated with 50 μM monastrol for 4 h to prevent the separation of spindle poles before being subjected to cold treatment and immunofluorescence staining. Cells with different levels of MT disassembly (as shown in Fig. 3c, e) were determined and counted.

### Analysis of phosphatidylinositol phosphate (PIP) binding with PLK1

The binding of each phosphatidylinositol phosphate (PIP) to PLK1 was assessed according to a previous study [41] with slight modifications. The PIP strips from Echelon Biosciences (P-6001, Salt Lake City, UT, USA) were first blocked in a solution of 3% (wt/vol) fatty acid-free BSA (A3803, Sigma) in PBS containing 0.1% Tween 20 (PBST) for 1 h at room temperature. The strip was then gently rinsed with PBST and incubated in blocking solution supplemented with 1 μg/ml recombinant human PLK1 (PV3501, Thermo Fisher Scientific Inc.) overnight at 4 °C with agitation. After removing the PLK1 solution and washing with PBST, the PIP strip was incubated with anti-PLK1 antibody (Invitrogen) followed by horseradish peroxidase (HRP)-conjugated secondary antibody (Jackson ImmunoResearch, West Grove, PA). PLK1 binding was detected by the enhanced chemiluminescence kit (Pierce Supersignal West Pico Plus Substrate; Rockford, IL).

### In vitro phosphorylation of MCAK by PLK1

MCAK was phosphorylated in vitro using cellular MCAK immunoprecipitated by specific antibody as a substrate and recombinant His-tagged PLK1 (Thermo Fisher Scientific Inc.) as an enzyme. Cellular MCAK was pulled down from 5 × 10^7^ cells using anti-MACK (Cytoskeleton, Inc.) and stored at − 80 °C in aliquots. The phosphorylation of MCAK was carried out with one tenth of the precipitated MCAK described above in 50 mM HEPES buffer containing 40 μM ATP, 10 mM MgCl_2_, 2 mM MnCl_2_, and 1 mM dithiothreitol. The reaction was initiated by addition of 100 ng of His-tagged PLK1, and the reaction mixture (30 μl) was then incubated in a water bath for 30 min at 30 °C. PI4P, PI5P, or PIP2 (Echelon Biosciences) was added into the reaction mixture at a final concentration of 10 μM. The reaction was then stopped by the addition of equal volumes of SDS-PAGE loading buffer, and proteins were subsequently separated by SDS-PAGE. The phosphorylation of MCAK was assessed using anti-phospho-serine (Cell Signaling) and HRP-conjugated secondary antibody, and the results were detected using an enhanced chemiluminescence kit, as described above.

## Data Availability

All data generated or analyzed during this study are included in the article.

## Abbreviations

KD: kinase domain
KIF: kinesin superfamily
K-MT: kinetochore microtubule
MCAK: mitotic centromere-associated kinesin
PI4P: phosphatidylinositol 4-phosphate
PI5P: phosphatidylinositol 5-phosphate
PIP2 (PI4,5P2): phosphatidylinositol 4,5-bisphosphate
PIP4KIIγ: phosphatidylinositol-5-phosphate 4-kinase type-2 gamma
PIP5KI: phosphatidylinositol 4-phosphate 5-kinase type I
PIP5Kiα: phosphatidylinositol 4-phosphate 5-kinase alpha
PIP4KII: phosphatidylinositol 5-phosphate 4-kinase type II
PIPK: phosphatidylinositol phosphate kinase
PBD: polo-box domain
PLK1: polo-like kinase
ROI: region of interest
